# Fisetin Inhibits Hyperglycemia-Induced Proinflammatory Cytokine Production by Epigenetic Mechanisms

**DOI:** 10.1155/2012/639469

**Published:** 2012-12-20

**Authors:** Hye Joo Kim, Seong Hwan Kim, Jung-Mi Yun

**Affiliations:** ^1^Pharmacology Research Center, Korea Research Institute of Chemical Technology, Daejeon 305-600, Republic of Korea; ^2^Department of Food and Nutrition, Kwangju Women's University, 165 Sanjeong Dong, Gwangsan-Gu, Gwangju 506-713, Republic of Korea

## Abstract

Diabetes is characterized by a proinflammatory state, and several inflammatory processes have been associated with both type 1 and type 2 diabetes and the resulting complications. High glucose levels induce the release of proinflammatory cytokines. Fisetin, a flavonoid dietary ingredient found in the smoke tree (*Cotinus coggygria*), and is also widely distributed in fruits and vegetables. Fisetin is known to exert anti-inflammatory effects via inhibition of the NF-*κ*B signaling pathway. In this study, we analyzed the effects of fisetin on proinflammatory cytokine secretion and epigenetic regulation, in human monocytes cultured under hyperglycemic conditions. Human monocytic (THP-1) cells were cultured under control (14.5 mmol/L mannitol), normoglycemic (NG, 5.5 mmol/L glucose), or hyperglycemic (HG, 20 mmol/L glucose) conditions, in the absence or presence of fisetin. Fisetin was added (3–10 *μ*M) for 48 h. While the HG condition significantly induced histone acetylation, NF-*κ*B activation, and proinflammatory cytokine (IL-6 and TNF-*α*) release from THP-1 cells, fisetin suppressed NF-*κ*B activity and cytokine release. Fisetin treatment also significantly reduced CBP/p300 gene expression, as well as the levels of acetylation and HAT activity of the CBP/p300 protein, which is a known NF-*κ*B coactivator. These results suggest that fisetin inhibits HG-induced cytokine production in monocytes, through epigenetic changes involving NF-*κ*B. We therefore propose that fisetin supplementation be considered for diabetes prevention.

## 1. Introduction

Diabetes and its complications are known for their chronic inflammatory properties. Inflammation is a defense reaction of host tissue against diverse insults. Although normally a beneficial process, when it occurs over prolonged periods, inflammation can be deleterious to cells [1]. Hyperglycemia has been implicated in diabetes-induced inflammatory disease and several diabetes-related complications [2, 3]. It has been reported to induce oxidative stress [4, 5]. In addition, high levels of glucose can activate the proinflammatory transcription factor nuclear *κ*B (NF-*κ*B), resulting in increased inflammatory chemokine and cytokine release [6, 7].

We and other researchers have recently shown that diabetic conditions activate inflammatory gene expression and monocyte activation by inducing epigenetic changes and chromatin remodeling [8, 9]. However, the exact molecular mechanisms induced by hyperglycemia are not fully resolved.

NF-*κ*B is required for the expression of many inflammatory genes. Several of these genes have been associated with inflammatory diseases, including atherosclerosis, insulin resistance, metabolic syndrome, and diabetes and its complications [10].

In mammals, the NF-*κ*B family contains 5 members: RelA/p65, RelB, c-Rel, NF-*κ*B1/p50, and NF-*κ*B2/p52. The classical NF-*κ*B transcription factor is a p50-RelA/p65 heterodimer [11], which regulates transcription of a number of inflammatory genes. NF-*κ*B activity is regulated by RelA/p65 acetylation and deacetylation, which are mediated by histone acetyltransferases (HATs) and histone deacetylases (HDACs), respectively [12, 13]. In addition to regulating NF-*κ*B activity, HATs and HDACs regulate inflammatory processes by acetylation/deacetylation of histones [14, 15].

The term “epigenetic” is used to describe heritable changes in phenotype or gene expression caused by mechanisms that do not involve modulation of DNA sequence [16]. Recently, epigenetic modifications have also been implicated in disease-associated changes in gene expression [17].

The best understood types of epigenetic regulation are DNA methylation and histone posttranslational modification. Histones can be subject to a number of different posttranslational modifications, among which acetylation and deacetylation have been extensively studied. In general, addition of acetyl groups to histones by HATs results in an “open” chromatin conformation, facilitating gene expression by allowing transcription factors to access DNA. In contrast, removal of acetyl groups by HDACs results in a “closed” chromatin environment, which has a repressive effect on transcription [18]. Thus, it is obvious that a tightly regulated balance between the activities of these 2 antagonistic enzymes is required for proper gene expression, while disruption of this balance can result in pathological conditions [19].

Altered HAT and HDAC activities can lead to several diseases, including cancer, diabetes, cardiac hypertrophy, and asthma [14, 15, 20, 21]. Recently, the *in vivo* relevance of histone acetylation in diabetes and inflammation was supported by a study demonstrating increased levels of histone lysine acetylation on inflammatory gene promoters in monocytes isolated from both type 1 and type 2 diabetes patients, relative to healthy control volunteers [9]. Other studies have addressed targeting of epigenetic mechanisms as a putative therapeutic means in the treatment of diabetes, primarily focusing on the use of small molecule HDAC modulators of natural origin [6, 7, 20–22]. However, the mechanisms of action of such molecules remain largely unknown.

Flavonoids are natural molecules examined as putative anti-inflammatory agents. They are low-molecular-weight polyphenolic compounds abundantly found in seeds, citrus fruits, red wine, tea, and olive oil. Flavonoids have diverse biological properties: in addition to their anti-inflammatory function, they have been described to exert antioxidant, antiplatelet, antithrombotic, cytoprotective, antiallergic, antiviral, and anticarcinogenic effects [23–26]. Due to their abundance in dietary products and their potential beneficial pharmacological and nutritional effects, flavonoids are of considerable interest both as drugs as well as health food supplements. Fisetin (3,7,3′,4′-tetrahydroxyflavone) is a flavonoid dietary ingredient found in the smoke tree (*Cotinus coggygria*) and is also widely distributed in fruits and vegetables, such as strawberry, apple, persimmon, grape, onion, and cucumber. It exhibits various activities, including neurotrophic, antioxidant, anti-inflammatory, and antiangiogenic effects [23, 27–29]. Fisetin has also been reported to downregulate glycogenolysis, gluconeogenesis, and formation of glycated hemoglobin *in vitro* [30, 31]. However, to date, the molecular mechanism of fisetin action remains unknown.

In the current study, we sought to address the use of fisetin as an anti-inflammatory agent, analyzing its molecular mechanism of action under diabetic conditions. We hypothesized that fisetin suppresses proinflammatory cytokine secretion through the NF-*κ*B signaling pathway, by altering the balance between histone acetylation and deacetylation. To test our hypothesis, we used human monocytes cultured under high-glucose conditions and analyzed the effect of fisetin on HAT and HDAC activity, NF-*κ*B acetylation, and inflammatory gene expression.

## 2. Materials and Methods

### 2.1. Reagents

Fisetin was purchased from Sigma Aldrich (St Louis, MI, USA). Fisetin was kept as a stock solution in Dimethyl Sulfoxide (DMSO) and was diluted with culture medium. We used 0.1% (v/v) DMSO as a vehicle control in all experiments. Real-time PCR primers were purchased from Bioneer (Daejeon, Korea). Antibodies against NF-*κ*Bp65, phosphorylated NF-*κ*Bp65, acetylated p65, p300, and acetylated CBP/p300 were purchased from Cell Signaling Technology (Beverly, MA, USA). Antibodies against HDAC-1, HDAC-2, and HDAC-3 were purchased from Abcam (Cambridge, MA, USA). Tumor necrosis factor *α* (TNF-*α*) and IL-6 ELISA assay kits were also purchased from Abcam (Cambridge, MA, USA). HAT and HDAC assay kits were purchased from Biovision (Mountain View, CA). The BCA protein assay kit was purchased from Pierce. Novex precast Tris-Glycine gels were obtained from Invitrogen (Carlsbad, CA, USA). All other chemicals, unless otherwise stated, were obtained from Sigma (St. Louis, MO, USA).

### 2.2. Cell Culture

The human monocytic THP-1 cell line was obtained from American Type Culture Collection (Manassas, VA). THP-1 cells were cultured in RPMI medium containing 10% fetal bovine serum and 1% antibiotics at 37°C in a 5% CO_2_ atmosphere. Fisetin dissolved in DMSO was used for treatment of the cells. The final concentration of DMSO used was 0.1% (v/v) for each treatment. THP-1 cells (1 × 10^5^ cells/mL) were cultured in the presence of osmolar control (14.5 mmol/L mannitol), under normal glycemic (NG, 5.5 mmol/L glucose), or hyperglycemic (HG, 20 mmol/L glucose) conditions, in the absence or presence of fisetin (0, 3, 6, 10 *μ*M) for 48 h. Following this period, the medium was collected for measurement of cytokine release; cells were washed with phosphate-buffered saline (PBS) and then harvested.

### 2.3. Cell Viability Assay

The toxic effects of fisetin on cultured THP-1 cells was measured by Cell Counting Kit-8 (Dojindo Molecular Technologies, ML, USA), according to the manufacturers protocol. THP-1 cells were seeded at 4 × 10^3^ cells/well in a 96-well plate and subsequently treated with fisetin for 48 h. Absorbance was measured using a Wallac EnVision microplate reader (PerkinElmer, Finland). The inhibitory effect of fisetin on growth was assessed as the percentage of cell growth reduction compared to vehicle-treated cells, which were defined as 100% viable.

### 2.4. Cytokine Release Measurement

Cytokine levels were measured using ELISA assay kits (Abcam, Cambridge, MA, USA) according to the manufacturer's instructions. Values were calculated based on a standard curve constructed for the assay.

### 2.5. Evaluation of mRNA Levels

Primers were designed using an online program. Total RNA was isolated using TRIzol reagent (Life Technologies, MD), according to the manufacturers' protocol. The concentration and purity of total RNA were assessed by measuring absorbance at 260 and 280 nm. First-strand cDNA was synthesized starting from 2 *μ*g of total RNA, using 1 *μ*M of oligo-dT_18_ primer and Omniscript Reverse Transcriptase (Qiagen, CA). SYBR green-based quantitative PCR was performed with the Stratagene Mx3000P Real-Time PCR system and Brilliant SYBR Green Master Mix (Stratagene, CA), using 3 *μ*L of first-strand cDNA diluted 1 : 50 as a template, and 10 pmoles of primers, according to the manufacturer's protocols. The PCR reaction consisted of three segments: the first segment (95°C for 10 min) activated the polymerase; the second segment included 35 cycles, each consisting of 40 s of denaturation at 94°C, followed by 40 s of annealing at 60°C, and 1 min of extension at 72°C; the third segment was performed to generate temperature dissociation curves of the products, by incubation at 95°C for 1 min, followed by incubation for 30 s at 55°C and 30 s at 95°C. All reactions were run in triplicate, and data were analyzed by the 2^−ΔΔCT^ method [32]. GAPDH was used as a normalization control gene. Significance was determined by comparison with GAPDH-normalized 2^−ΔΔCT^ values.

### 2.6. Measurement of HDAC and HAT Activity Using ELISA

Following treatment with various concentrations of fisetin for 48 h, cells were harvested and nuclear lysates were prepared. For determination of HAT and HDAC activity, nuclear lysate containing 50 *μ*g of protein was taken from each group. The experiment was performed according to the manufacturer's instructions. Absorbance was measured at 405 nm and 440 nm.

### 2.7. Preparation of Nuclear and Cytoplasmic Lysates

After treatment with fisetin, the medium was aspirated and cells were washed twice in PBS (10 mM, pH 7.4). Nuclear lysates were prepared using NE-PER Nuclear and Cytoplasmic Extraction Reagents (Pierce, IL, USA). Lysates were collected and cleared by centrifugation, and the supernatant was aliquoted and stored at −80°C. The protein concentration of the lysates was measured by BCA protein assay (Pierce, IL, USA), as per the manufacturer's protocol.

### 2.8. Western Blot Analysis

For western blot analysis, cells were homogenized in buffer consisting of 10 mM Tris-HCl (pH 7.5), 150 mM NaCl, 0.05% (v/v) Tween 20, 1 mM PMSF, and one protease inhibitor cocktail tablet (Roche, Germany) at 4°C, and then centrifuged at 10,000 ×g for 15 min. The supernatant was used as the cytoplasmic protein fraction, and nuclear proteins were extracted from the pellet using the NucBuster Protein Extraction kit (Novagen, Germany). Protein concentration was determined using the BCA protein assay kit (Pierce, IL). Samples (20 *μ*g of total protein) were mixed with sample buffer (100 mM Tris-HCl, 2% sodium dodecyl sulfate, 1% 2-mercaptoethanol, 2% glycerol, and 0.01% bromophenol blue (pH 7.6), incubated at 95°C for 15 min, and loaded on 10% polyacrylamide gels. Electrophoresis was performed using the Mini Protean 3 Cell system (Bio-Rad, CA). The resolved proteins were transferred on a nitrocellulose membrane (Scheicher & Schnell BioScience, Germany). To visually assess the amount of protein loaded and the efficiency of the transfer, membranes were stained with Ponceau S staining solution. For immunoblotting, membranes were washed and incubated in blocking buffer (10 mM Tris-HCl pH 7.5, 150 mM NaCl, 0.1% Tween 20, and 3% nonfat dry milk), and then incubated with diluted primary antibodies (1 : 1000) for 2 h at room temperature. Following incubation with the primary antibody, membranes were washed 3 times with blocking buffer and then probed with diluted secondary antibodies (1 : 2000) for 1 h. The membranes were washed 3 times (15 min each) and developed with SuperSignal West Femto Maximum Sensitivity Substrate (Pierce, IL, USA), using a LAS-3000 luminescent image analyzer (Fuji Photo Film Co. Ltd., Japan).

### 2.9. Immunoprecipitation Assays

For immunoprecipitation, we isolated the nuclear fraction of cells, as per the manufacturer's protocol. Samples (300 *μ*g of total protein) were precleared with protein A/G plus agarose (Santacruz Biotechnology Inc., CA USA) for 1 h at 4°C. After 1 h, the supernatant fraction of each sample was transferred to a fresh tube and incubated with the respective antibody (2 *μ*g/mL) overnight at 4°C. Following incubation with the antibody, samples were mixed with 40 *μ*L of protein A/G plus agarose for 2 h at 4°C. The samples were subject to microcentrifugation and then washed 3 times with PBS. Following the washing steps, samples were mixed with 15 *μ*L of 2x SDS sample buffer and then subjected to western blot analysis.

### 2.10. Chromatin Immunoprecipitation Assays

ChIP assays were performed using MAGnify ChIP according to the manufacturer's instructions. After treatment of cells with fisetin, the cells were centrifuged and medium was aspirated. Cells were washed twice in PBS (10 mM, pH 7.4) and fixed with fresh Fixation solution (37% formaldehyde) for 10 min at room temperature, followed by glycine stop-fix solution. Cells were washed twice with cold PBS, PBS was poured off and discarded and cells were scraped, pelleted by centrifugation for 10 min at 7,000 rpm at 4°C. Cells were resuspended in 100 *μ*L of ice-cold lysis buffer followed by 5 min incubation on ice. Pellets were spun down for 10 min at 5000 rpm. Chromatin was sheared using high power sonication using our optimized condition (16 cycle; 30 pulses of 30 s each with a 30-s rest on ice between pulses) to an average DNA size of 500 bp and lysates were cleared by centrifugation at 13,000 for 10 min at 4°C. For each ChIP, one-tenth of the total sonicated chromatin volume (100 *μ*L) was used. Immunoprecipitations were performed overnight at 4°C with 5 *μ*g of the p300 antibody. Chromatin-antibody complexes were captured to magnetic beads (20 *μ*L), and chromatin was eluted as described in manufacturer's instructions. The cross-links were reversed and DNA was purified by proteinase K. DNA was analyzed by PCR.

### 2.11. PCR

DNA concentration was measured spectrophotometrically at 260 nm. DNA was subjected to PCR. The antibodies against p300 were purchased from Santa Cruz Biotechnology. Primer sequences for the amplification of tumor necrosis factor *α* (TNF-*α*) were: forward: 5′-CCTCCCAGTTCTAGTTCTATC-3′ and reverse: 5′-GGGGAAAGAATCATTCAACCAG-3′. PCR was performed after a 5-minute denaturation at 94°C, and repeating the cycles of 94°C, 55°C for each 30 s and 72°C for 45 s; the 35 of cycles were specific for primer set. PCR products were electrophoresed in a 1.5% agarose gel containing ethidium bromide.

### 2.12. Statistical Analysis

Each experiment was performed at least 3 times. Results are expressed as the mean value ± standard deviation (SD). Statistical analysis was performed using Student's *t*-test, and statistical significance was set at *P* < 0.05 for some analyses and *P* < 0.01 for others. They have been separately indicated in the figures.

## 3. Results

### 3.1. Toxic Effects of Fisetin on Monocytes under Hyperglycemia

**Figure 1:**
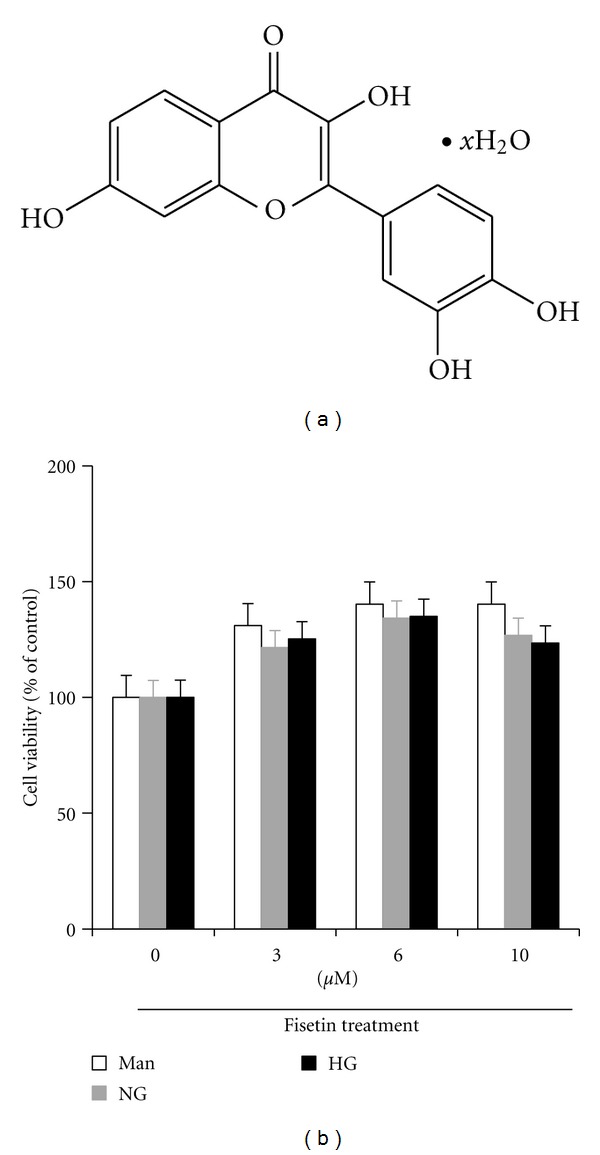
The cytotoxicity of fisetin to cultured high glucose-induced THP-1 cells. (a) Chemical structure of fisetin. (b) Effect of fisetin on cell viability after 48 h was evaluated by the CCK-8 assay, as described in the methods. Human monocytic (THP-1) cells (1 × 10^5^ cells/mL) were cultured in presence of osmolar control (14.5 mmol/L mannitol) or normal glycemic (NG, 5.5 mmol/L glucose) or hyperglycemic (HG, 20 mmol/L) conditions in absence or presence of fisetin (0, 3, 6, 10 *μ*M) for 48 h as described in the methods and the media was collected. Results are shown as mean ± SD of five different experiments.

The chemical structure of fisetin is shown in Figure 1(a). We investigated the cytotoxic effect of fisetin on high glucose-induced THP-1 cells, using CCK-8 assay (Figure 1(b)). No toxicity was observed at concentrations of fisetin between 3 and 10 *μ*M, for 48 h of treatment. All our experiments were performed in the latter, nontoxic concentration range of fisetin (Figure 1(b)).

### 3.2. Effects of Fisetin on Proinflammatory Cytokine Secretion in Monocytes under Hyperglycemic Conditions

**Figure 2:**
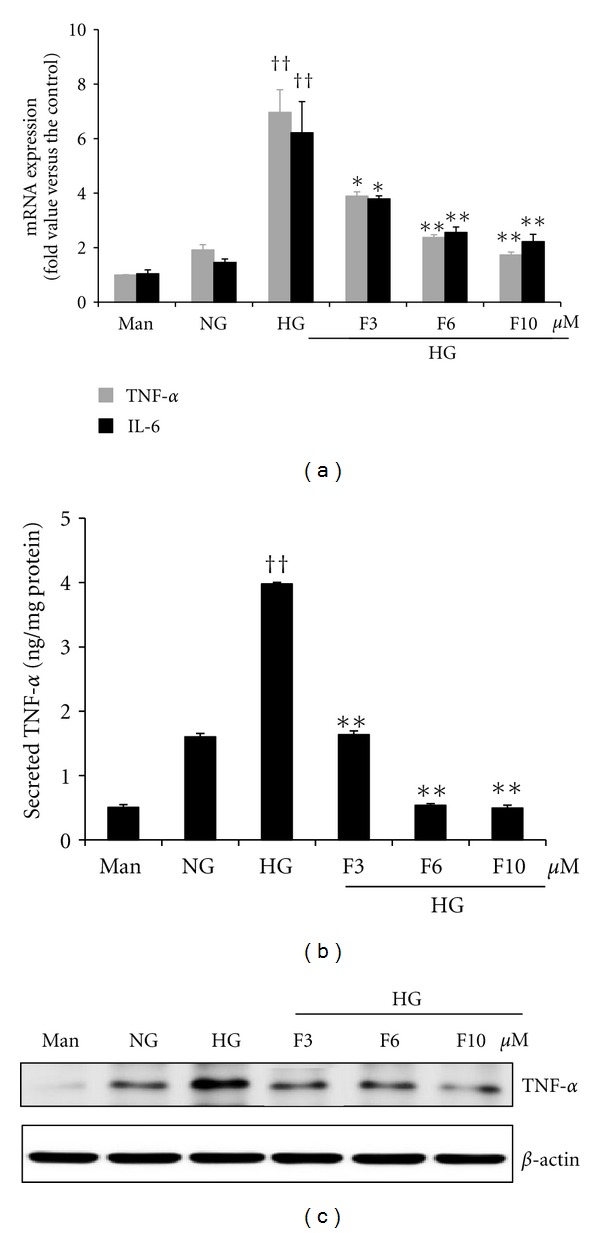
Fisetin-mediated inhibition of cytokine release in HG-treated THP-1 cells. (a) Cells (1 × 10^5^ cells/mL) were treated with fisetin for 48 h and then mRNA levels were evaluated by quantitative real-time PCR. (b) Cell media were collected for TNF-*α* measurement by ELISA assay kit. Cytokine levels in the media were measured with ELISA assay kit according to the manufacturer's instructions. Values were calculated based on a standard curve constructed for the assay. Results are shown as mean ± SD of five different experiments. ^††^ *P* < 0.01 compared to NG; *P < 0.05; **P < 0.01 compared to HG. (c) Cell lysates were prepared and TNF-*α* level was evaluated by western blot analysis as described in the methods. Equal loading of protein was confirmed by stripping the immunoblot and reprobing it for *β*-actin protein. The immunoblots shown here are representative of 3 independent experiments.

We examined whether fisetin could inhibit proinflammatory cytokine genes, such as TNF-*α* and IL-6, in high-glucose-treated THP-1 cells. Under hyperglycemic conditions, inflammatory cytokine release was significantly increased compared to under normal glycemic conditions. Mannitol was used as a hyperosmolar control and did not affect cytokine release. As shown in Figure 2(a), treatment of fisetin significantly inhibited high glucose-induced mRNA expression levels of TNF-*α* and IL-6. To confirm the effect of fisetin on the expression of proinflammatory cytokines, culture media were assayed for TNF-*α* levels by ELISA, and nuclear lysates were subjected to western blot assay. As shown in Figures 2(b) and 2(c), fisetin significantly decreased the secretion of cytokine, TNF-*α*, under hyperglycemic conditions in human monocytes.

### 3.3. Modulatory Effect of Fisetin on HAT and HDAC Activity in Monocytes under Hyperglycemic Conditions

**Figure 3:**
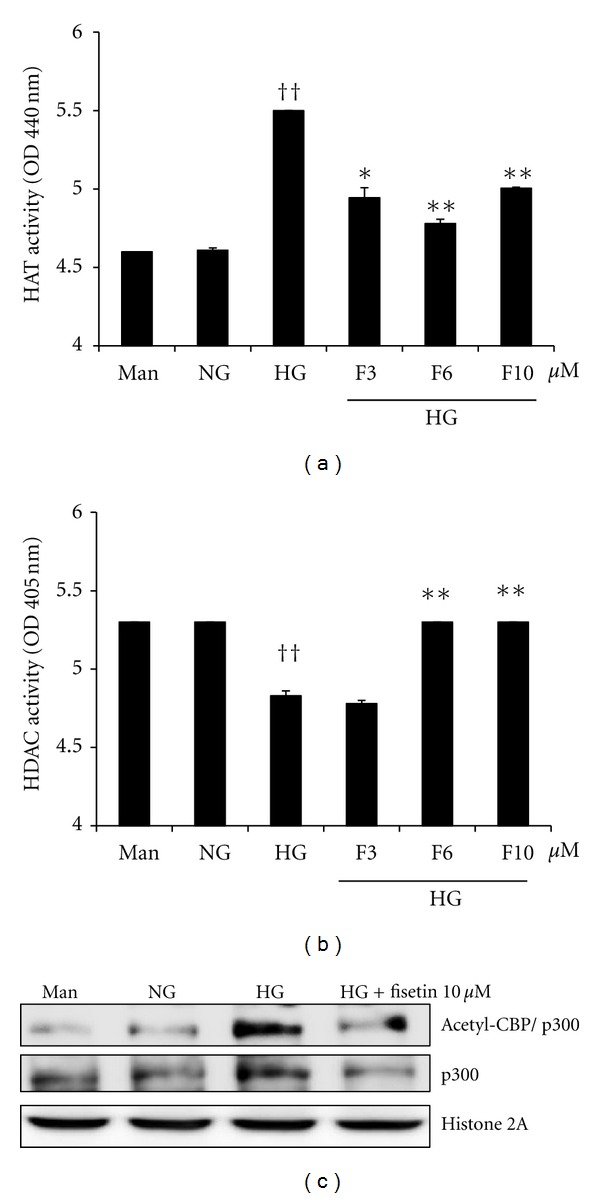
Effect of fisetin on HAT and HDAC activity as well as p300 and acetylated CBP/p300 levels in HG-treated THP-1 cells. Cells were harvested after 48 h of fisetin treatment and nuclear lysates were prepared. Samples were analyzed for determination of HAT (a) and HDAC activity (b). Results are shown as mean ± SD for 3 different experiments. ^††^ *P* < 0.01 compared to NG; *P < 0.05; **P < 0.01 compared to HG. (c) After nuclear protein extraction, p300 and acetylated CBP/p300 levels were evaluated by western blot. The immunoblots shown here are representative of 3 independent experiments.

We next addressed the mechanism by which fisetin inhibits cytokine gene expression in monocytes. To obtain further insights into the mechanisms of fisetin-induced downregulation of inflammatory cytokines, we first examined whether fisetin treatment modulates HAT and HDAC activity, using ELISA. As shown in Figure 3, under hyperglycemic conditions, there was a significant increase in HAT activity and decrease in HDAC activity compared to normal glucose conditions (*P* < 0.01). Interestingly, fisetin treatment results in a significant downregulation of HAT and upregulation of HDAC activity (*P* < 0.01). High glucose levels activate transcription factors, such as NF-*κ*B, by recruitment of transcriptional coactivator molecules CBP/p300, which possess intrinsic HAT activity. The resulting increase in histone acetylation and DNA unwinding allow RNA polymerase to access DNA, leading to proinflammatory gene expression. As shown in Figure 3(c), THP-1 cells cultured under hyperglycemic conditions showed marked upregulation of p300 as well as its acetylation levels, compared with cells cultured under normal glycemic conditions. d-mannitol had no effect on p300. As shown Figure 3(c), p300 activation was abolished by fisetin (10 *μ*M) treatment. Fisetin also decreased the levels of acetylated CBP/p300 in high-glucose conditions, to levels comparable to those observed under normal glucose conditions. No effects were observed with DMSO (0.1%) vehicle control treatment.

### 3.4. Effect of Fisetin on NF-*κ*B p65 Activation in Monocytes under Hyperglycemic Conditions

**Figure 4:**
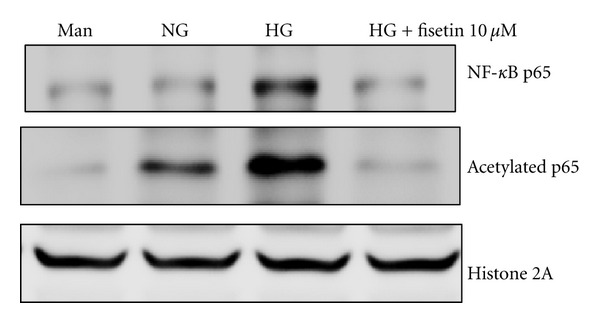
Fisetin-induced suppression of NF-*κ*B activation in HG-treated THP-1 cells. Protein levels were evaluated by western blot for NF-*κ*B p65 and acetylated p65. Equal loading of protein was confirmed by stripping the immunoblot and reprobing it for histone 2A protein. The immunoblots shown here are representative of 3 independent experiments.

Histone acetylation is associated with increased NF-*κ*B activation that leads to increased acetylation of the RelA/p65 subunit of NF-*κ*B [3]. Therefore, we studied the effect of fisetin on acetylation of p65 and subsequent NF-*κ*B activation, under high-glucose conditions. We observed that fisetin resulted in significantly decreased acetylation and phosphorylation of NF-*κ*Bp65 in the nuclear faction (Figure 4).

### 3.5. Effect of Fisetin on the Interaction of p300 with Inflammation-Associated Genes

**Figure 5:**
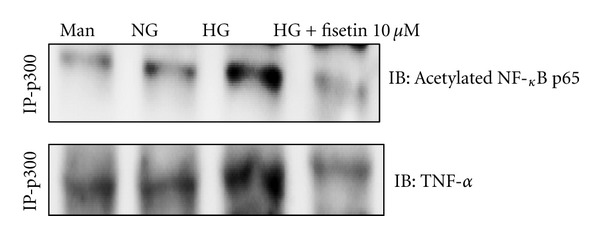
Effect of fisetin on the interaction of p300 with acetylated p65 and TNF-*α*. Cells were treated with fisetin for 48 h and then nuclear lysates were prepared. p300 was immunoprecipitated, and interaction with acetylated p65 and TNF-*α* was assessed by western blotting. The immunoblots shown here are representative of 3 independent experiments.

To further understand the mechanism of fisetin-mediated inhibition of inflammation, we investigated its effect on the interaction between p300 and NF-*κ*B. As shown in Figure 5, fisetin reduced the interaction of p300 with both the acetylated form of NF-*κ*B and with TNF-*α*. This was associated with decreased TNF-*α* gene transcription in monocytes under HG conditions.

### 3.6. Effect of Fisetin on Chromatin Events at the Promoters of Inflammatory Genes

**Figure 6:**
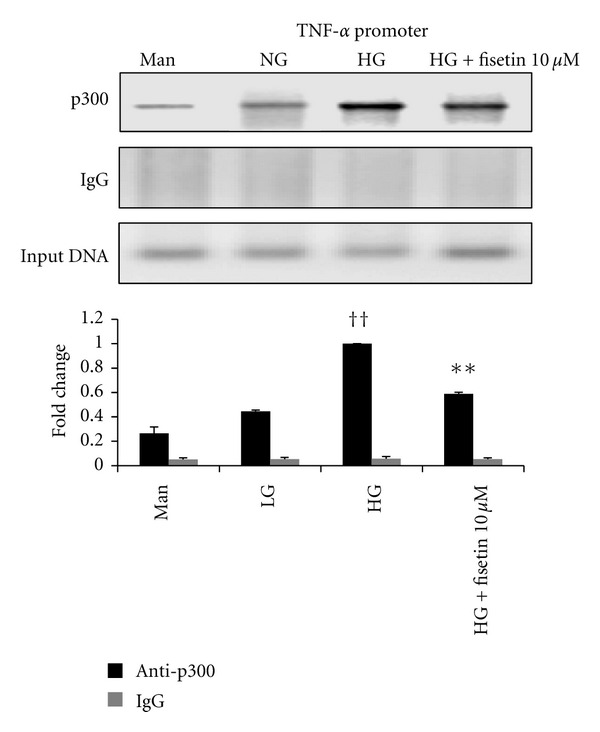
Effect of fisetin on chromatin event at the promoter of inflammatory gene. ChIP assays were performed using MAGnify ChIP according to the manufacturer's instructions. Immunoprecipitations were performed overnight at 4°C with 5 *μ*g of p300 antibody. DNA was subjected to PCR. ChIP assays showed the recruitment of p300 to the TNF-*α* promoters. Results of 1 typical experiment of 3 are shown. Values from ChIP with anti-p300 antibody represent the fold difference relative to those from IgG control antibody. ^††^ *P* < 0.01 compared to NG; *P < 0.05; **P < 0.01 compared to HG. Results are shown as mean ± SD for 3 different experiments.

To confirm the epigenetic regulation of fisetin on inflammation, we next used ChIP assays to further investigate whether p300 can be bound to the promoters of NF-*κ*B-related inflammatory cytokine genes under HG conditions. ChIP assays showed that HG increased the recruitment of p300 to the TNF-*α* promoter. As shown in Figure 6, Fisetin reduced the binding of p300 to the promoter region of TNF-*α*. This was associated with decreased TNF transcription in monocytes under HG conditions.

## 4. Discussion

Diabetes is a proinflammatory condition and chronic inflammation plays an important role in the progression of diabetic complications. Hyperglycemia has been implicated as a major contributor in several diabetes complications [2, 3].

THP-1 monocytes or human peripheral blood monocytes cultured under high-glucose conditions are a relevant cell culture model for the study of hyperglycemia. High glucose levels are known to induce expression of the inflammatory cytokine TNF-*α*, chemokines, and monocyte chemoattractant protein 1 (MCP-1), in these cells, in an oxidative stress-, NF-*κ*B-, and AP-1 transcription factor-dependent manner [6, 7]. We have recently shown that hyperglycemia induces proinflammatory cytokine release (IL-1, IL-6, and TNF-*α*) in monocytes via an NF-*κ*B-dependent pathway [6–8, 33, 34]. Systemic levels of proinflammatory cytokines, including TNF-*α*, IL-1*β*, and IL-6, are elevated in patients with both type 1 and type 2 diabetes [9, 34, 35]. In recent years, several clinical and animal studies have indicated that inflammatory cytokines play an important role in the development and progression of diabetic complications [36].

NF-*κ*B plays a crucial role in the expression of cytokines, including TNF-*α* and IL-6 [9, 35–39]. Schmid et al. have reported that NF-*κ*B plays a critical role in diabetes complications as it regulates transcription of a number of genes involved in inflammatory response [40]. Histone acetylation of NF-*κ*B target genes is generally associated with increased binding of the transcription factor to its response elements and active transcription [14]. HATs and HDACs play an important role in regulating proinflammatory response.

The antioxidant and/or anti-inflammatory effects of dietary polyphenols have been shown to play a role in either controlling NF-*κ*B activation or chromatin remodeling through modulation of HDAC and HAT activity, consequently affecting inflammatory gene expression [8, 18, 41–43].

Fisetin is a major flavonoid with a wide range of pharmacological effects, such as inhibition of angiogenesis, as well as anticancer, antiallergenic, and antithyroid activities [44–47]. Fisetin has been reported to downregulate both glycogenolysis and gluconeogenesis *in vitro* [30]. Furthermore, recent studies revealed hypoglycemic activity of fisetin in streptozotocin-induced experimental diabetes in rats [48, 49]. However, its specific regulation mechanisms at the chromatin level are not known in diabetic conditions.

The goal of this study was to determine whether fisetin can be used as a therapeutic agent for treatment of inflammation, which contributes to diabetes-related complications. We investigated the role of fisetin in regulation of high glucose-mediated proinflammatory cytokines (IL-6 and TNF-*α* release), HAT and HDAC modulation, and posttranslational modification of the transcription factor NF-*κ*B, in high-glucose-treated THP-1 cells.

Production of reactive oxygen species has been implicated as a causative factor for hyperglycemic damage [50–52]. Reactive oxygen species alter nuclear histone acetylation and deacetylation balance, leading to increased NF-*κ*B-dependent gene expression of proinflammatory mediators [51–53]. NF-*κ*B plays an important role in the regulation of proinflammatory genes that are associated with several inflammatory diseases, including atherosclerosis, insulin resistance, metabolic syndrome, and diabetes and its complications [9]. The p65 protein is a key component of NF-*κ*B activation and its transactivation potential is enhanced by several coactivators, including CREB-binding protein/p300, p/CAF and SRC1, which possess HAT activity [54–57]. In addition, acetylation of p300 at Lys1499 has been demonstrated to enhance its HAT activity and affect a wide variety of signaling events [58]. CBP/p300-mediated hyperacetylation of RelA is critical for NF-*κ*B activation. Five main acetylation sites have been identified on p65. Acetylation at Lys221 enhances DNA binding by p65 and inhibits its interaction with I*κ*B*α*, whereas acetylation of Lys316 is required for full transcriptional activity of p65 [57]. Accordingly, the attenuation of p65 acetylation is a potential molecular target for the prevention of chronic inflammation [9].

We found that fisetin treatment inhibited the expression of NF-*κ*B target genes, including IL-6 and TNF-*α*, in high-glucose-treated THP-1 cells. We also show novel data supporting fisetin-mediated inhibition of hyperglycemia-induced p65 acetylation, resulting in suppressed NF-*κ*B transcription activity. We also observed that fisetin can inhibit inflammation through upregulation of HDAC activity in HG-treated THP-1 cells.

In contrast, fisetin inhibited HAT activity, preventing NF-*κ*B-mediated chromatin acetylation and subsequent transcription of cytokines in hyperglycemic conditions. Fisetin also reduced p300 expression as well as its interaction with NF-*κ*B. Thus, fisetin appears to suppress inflammatory cytokines through at least in part the NF-*κ*B signaling pathway via inducing HDAC activity and suppressing HAT activity under HG conditions.

In summary, high glucose levels activate HAT (p300) and reduce HDAC activity, leading to increased acetylation of p65. Acetylation of p65, in turn, induces NF-*κ*B activation and transcription of IL-6 and TNF-*α* in monocytes. Fisetin treatment was cytotoxic at 30 *μ*M on monocytes under hyperglycemia (data not shown). We observed that 3–10 *μ*M is nontoxic and we used very effective concentration (0, 3, 6, 10 *μ*M) in this study. Administration of fisetin to cells cultured under hyperglycemic conditions may activate HDACs and suppress HATs, particularly p300, leading to deacetylation of the p65 subunit of NF-*κ*B. Thus, fisetin administration suppresses proinflammatory cytokine release. It may therefore be considered for use in diabetes preventions. Future studies are needed to uncover the specific chromatin events and molecular mechanisms induced by fisetin in hyperglycemic conditions.

## 5. Conclusion

In the current study, we hypothesized that fisetin suppresses proinflammatory cytokine secretion through the NF-*κ*B signaling pathway, by altering the balance between histone acetylation and deacetylation. Administration of fisetin to cells cultured under hyperglycemic conditions may activate HDACs and suppress HATs, particularly p300, leading to deacetylation of the p65 subunit of NF-*κ*B. Thus, fisetin administration suppresses proinflammatory cytokine release. To our knowledge, this is the first report for analyzing its molecular mechanism of action under diabetic conditions. Understanding these mechanisms will be critical towards establishment of fisetin as a natural therapeutic agent for the treatment of chronic inflammation associated with diabetes and its complications.
